# Disease-specific regulation of gene expression in a comparative analysis of juvenile idiopathic arthritis and inflammatory bowel disease

**DOI:** 10.1186/s13073-018-0558-x

**Published:** 2018-06-27

**Authors:** Angela Mo, Urko M. Marigorta, Dalia Arafat, Lai Hin Kimi Chan, Lori Ponder, Se Ryeong Jang, Jarod Prince, Subra Kugathasan, Sampath Prahalad, Greg Gibson

**Affiliations:** 1 Center for Integrative Genomics and School of Biological Sciences, Georgia Institute of Technology, Engineered Biosystems Building, EBB 2115, 950 Atlantic Drive, Atlanta, GA 30332 USA; 2 Department of Pediatrics, Emory University School of Medicine and Children’s Healthcare of Atlanta, 1760 Haygood Dr NE, Atlanta, GA 30322 USA

**Keywords:** Juvenile idiopathic arthritis, Inflammatory bowel disease, eQTL, Gene expression

## Abstract

**Background:**

The genetic and immunological factors that contribute to differences in susceptibility and progression between sub-types of inflammatory and autoimmune diseases continue to be elucidated. Inflammatory bowel disease and juvenile idiopathic arthritis are both clinically heterogeneous and known to be due in part to abnormal regulation of gene activity in diverse immune cell types. Comparative genomic analysis of these conditions is expected to reveal differences in underlying genetic mechanisms of disease.

**Methods:**

We performed RNA-Seq on whole blood samples from 202 patients with oligoarticular, polyarticular, or systemic juvenile idiopathic arthritis, or with Crohn’s disease or ulcerative colitis, as well as healthy controls, to characterize differences in gene expression. Gene ontology analysis combined with Blood Transcript Module and Blood Informative Transcript analysis was used to infer immunological differences. Comparative expression quantitative trait locus (eQTL) analysis was used to quantify disease-specific regulation of transcript abundance.

**Results:**

A pattern of differentially expressed genes and pathways reveals a gradient of disease spanning from healthy controls to oligoarticular, polyarticular, and systemic juvenile idiopathic arthritis (JIA); Crohn’s disease; and ulcerative colitis. Transcriptional risk scores also provide good discrimination of controls, JIA, and IBD. Most eQTL are found to have similar effects across disease sub-types, but we also identify disease-specific eQTL at loci associated with disease by GWAS.

**Conclusion:**

JIA and IBD are characterized by divergent peripheral blood transcriptomes, the genetic regulation of which displays limited disease specificity, implying that disease-specific genetic influences are largely independent of, or downstream of, eQTL effects.

**Electronic supplementary material:**

The online version of this article (10.1186/s13073-018-0558-x) contains supplementary material, which is available to authorized users.

## Background

While genomic analyses have clearly established a high degree of shared genetic susceptibility across autoimmune and inflammatory disorders, the reasons for disease-specific effects of particular loci are yet to be understood [1]. Likely explanations range from the technical, such as variable statistical power across studies, to the biological, including restriction of effects to relevant cell types for each condition, and interactions between genotypes and either the environment or genetic background. Since the majority of genome-wide association study (GWAS) associations are likely regulatory, attention has focused on mapping genetic effects on gene expression and/or epigenetic marks, namely discovery of expression quantitative trait locus (eQTL) and their methylation counterparts, mQTL [2]. With a few exceptions, most studies attempting to relate GWAS to functional genomics have utilized large public eQTL and epigenetic datasets of peripheral blood-derived profiles of healthy volunteers. These implicitly assume equivalence of eQTL across health and disease, despite recent findings that eQTL can be modified by ex vivo treatments which mimic perturbations corresponding to disease states [3, 4]. In order to evaluate the ratio of common to disease-specific effects in inflammatory autoimmune disease, here we describe side-by-side comparative eQTL analysis of juvenile idiopathic arthritis (JIA) and inflammatory bowel disease (IBD), also comparing the transcriptomes among major sub-types within both JIA and IBD.

IBD has been extensively studied using a variety of genomic approaches, but despite several early publications, JIA has been less well characterized [5–8]. JIA is the most common rheumatic disease of childhood, with an estimated prevalence of approximately 1.2 individuals per 1000 in the USA [9]. It comprises multiple clinically and genetically distinct forms of arthritis with onset prior to age 16. Although all forms of JIA are characterized by persistent swelling of the joints, the disease is further classified into sub-types based on clinical presentation [10]. Oligoarticular JIA affects four or fewer joints and is the most common and typically the mildest form of JIA [10, 11]. Polyarticular JIA involves five or more joints and is intermediate in severity. Both oligoarticular and polyarticular JIA disproportionately affect females. Systemic JIA (sJIA) is distinct from other JIA sub-types, displaying unique symptoms and no bias towards females [10, 12]. Diagnosis is based on presentation of arthritis accompanied by spiking fever, rash, and lymphadenopathy. Approximately 10% of sJIA patients are also diagnosed with life-threatening macrophage activation syndrome, and about 50% experience a persistent course of disease and are unable to achieve remission [12, 13].

The categorization of sub-types based primarily on clinical criteria reflects uncertainty about the biological factors that contribute to the heterogeneity of the disease. The immune system is thought to play a critical role in the pathogenesis of JIA. Levels of immune-related cells like lymphocytes, monocytes, and neutrophils are differentially elevated between sub-types [14], as is also seen in other autoimmune and autoinflammatory diseases such as rheumatoid arthritis (RA) and inflammatory bowel disease [15]. Evidence of T cell activation has been described in oligoarticular and polyarticular patients, suggesting the importance of adaptive immunity in these sub-types [11, 16], but there is considerable heterogeneity in immune profiles that masks differences between levels of severity [17, 18], with age-of-onset also an important factor influencing gene expression [19]. In contrast, sJIA is thought to be more characterized by activation of innate immunity and upregulated monocytes, macrophages, and neutrophils [12, 20].

Extensive genome-wide association studies have been performed across autoimmune classes and are conveniently summarized on the ImmunoBase website, which as of February 2018 lists 23 validated loci for JIA, 81 for RA, 102 for ulcerative colitis (UC), and 122 for Crohn’s disease (CD) [21]. Previous studies have demonstrated familial aggregation of JIA, supporting the idea that genetics plays a role in susceptibility [22] as well as sub-type development. Studies of genetic variants within the major histocompatibility complex region have uncovered associations between various human leukocyte antigen (HLA) polymorphisms and sub-types of JIA [23, 24]. HLA-independent loci such as *PTPN22* and *STAT4* have also been repeatedly found in genome-wide association studies to be associated with oligoarticular and RF-negative polyarticular JIA at genome-wide significance levels [25–28], while polymorphisms in interleukins 1 and 10 were early on identified as occurring at higher frequencies in sJIA patients [29, 30]. The most recent international GWAS of 982 children with sJIA concluded that the systemic form of JIA engages more inflammatory than autoimmune-related genes [31], consistent with clinical observations of the course of disease.

Diverse autoimmune conditions certainly are attributable in part to intrinsic aspects of the focal tissue and in part to gene activity in the immune system, some of which should be detectable in peripheral blood samples. It is thus surprising that side-by-side comparisons of immune gene expression across disease sub-types have not been reported. Transcriptomic studies of disease are for practical reasons orders of magnitude smaller than GWAS, typically involving fewer than 200 patients, but these are nevertheless sufficient to identify eQTL given the relatively large effect of regulatory polymorphisms on local gene expression. Numerous blood- and tissue-specific susceptibility loci and eQTL have previously been discovered [32–34]. It is likely that sJIA in particular shares associated risk polymorphisms with IBD given the auto-inflammatory component of both diseases. For instance, a mutation in *LACC1* that was initially associated with Crohn’s disease was later found also to be associated with sJIA [35, 36]. Thus, IBD is an attractive candidate for comparison with JIA to elucidate the mechanisms behind each of the sub-types. Here we contrast healthy controls; patients with oligoarticular, polyarticular, or systemic JIA; and patients with two forms of IBD, CD, or UC. As well as evaluating overall transcriptome differences among sub-types, we evaluate the disease specificity of whole blood eQTL effects in order to infer what fraction of risk can be attributed to differences in genetic regulation of gene expression.

## Methods

### Cohorts

In total, there were 190 patients and 12 controls. Protocols including signed consent of all participants and/or assent of parents in the case of minors were approved by the IRBs of Emory University and Georgia Institute of Technology. All patient cohorts were comprised of individuals of European (*n* = 141) or African (*n* = 49) ancestry from the USA. The cohorts are further divided into IBD and JIA subgroups. Within the IBD subgroup, 60 individuals were CD patients while 15 were UC patients. The average age of disease onset for CD and UC patients was approximately 14 years, with ages of onset ranging from less than 1 to 26 years. The JIA subgroup was comprised of 43 oligoarticular, 46 polyarticular, and 26 systemic JIA patients. The average age of disease onset for JIA patients was 8 years, with onset ages ranging from 0.7 to 17 years.

### RNA-Seq processing and differential gene expression analysis

RNA was isolated from whole blood, and RNA-Seq was used to determine profiles of gene expression. The paired-end 100 bp reads were mapped to human genome hg19 using TopHat2 [37] with default parameters, with 90.4% success rate. The aligned reads were converted into number of reads per gene using SAMtools and HTSeq with the default union mode [38, 39]. The raw counts were then processed by trimmed mean of *M*-values normalization via the edgeR R package into normalized counts [40]. To further normalize and remove batch effects from gene expression data, surrogate variable analysis (SVA) combined with supervised normalization was used [41]. First, FPKM was calculated and all genes with greater than 10 individuals with greater than six read counts and FPKM > 0.1 were extracted. Expression of the sex-specific genes RPS4Y1, EIF1AY, DDX3Y, KDM5D, and XIST was used to verify the gender of each individual. The SVA R package [41] was used to identify 15 latent confounding factors, and these were statistically removed without compromising known disease variables using the supervised normalization procedure in the SNM R package [42]. Pairwise comparisons between control, CD, UC, oligoarticular JIA, polyarticular JIA, and systemic JIA were performed to quantify the extent of differential expression. Using edgeR’s generalized linear model likelihood ratio test function, the log fold change and Benjamini-Hochberg adjusted *p* value were obtained for all genes within each contrast [40].

Gene ontology analysis was performed using the GOseq R package, which incorporates RNA-Seq read length biases into its testing [43]. Genes with an edgeR-calculated FDR of < 0.01 were considered to be differentially expressed and input into the GOseq software. Genes were distinguished by positive and negative log fold change to classify upregulation in specific sub-types. Only pathways within the biological processes and molecular function gene ontology branches were called.

Analysis of established immune-related gene sets was performed using BIT (Blood Informative Transcript) and BTM (Blood Transcript Module) gene expression [44, 45]. The BITs are highly co-regulated genes which define seven axes of blood immune activity that are highly conserved across whole blood gene expression datasets. Standard PCA analysis including multiple PC captures most of the variance also described by the BIT, but it does so in a study-specific manner in which the actual PC have little biological meaning. By contrast, the BIT axes, as originally characterized by Preininger et al. [44], capture components of variation that are consistently observed across all peripheral blood gene expression studies, for the most part independent of platform. We simply take PC1 for the representative genes for each axis and note that this typically explains upwards of 70% of variance of those transcripts, so it is highly representative of overall gene expression in the axis. Whereas in previous work [44] we labelled nine axes BIT axis 1 through 9, subsequent analyses and comparison with BTMs has led to affirmation of the immunological functions captured by six of the axes, which we here rename reflecting these functions as axis T (T cell-related, formerly 1), axis B (B cell-related, formerly 3), axis N (neutrophil-related, formerly 5), axis R (reticulocyte-related, formerly 2), axis I (interferon-responsive, formerly 7), and axis G (general cellular biosynthesis, formerly 4). axis 6 remains of uncertain function, while axes 8 and 9 are dropped since they are derivative and less consistent. Finally, a newly identified axis C captures numerous cell cycle-related aspects of gene activity. Each of these axes clusters with a subset of the 247 BTMs identified by Li et al. in their machine-learning meta-analysis of 30,000 peripheral blood gene expression samples from over 500 studies [45], and these relationships were visualized by hierarchical cluster analysis performed using Ward’s method in SAS/JMP Genomics [46].

### SNP data processing and eQTL analysis

The Affymetrix Axiom BioBank and Illumina Immunochip arrays were used to perform genotyping, at Akesogen Inc. (Norcross, GA). Quality control was performed using PLINK, with parameters set to remove non-biallelic variants, SNPs not in Hardy-Weinberg equilibrium at *P* < 10^−3^, minor allele frequency < 1%, and rate of missing data across individuals > 5% [47].

The Affymetrix Axiom BioBank array, which has a coverage of 800 k SNPs, was utilized to genotype the 115 JIA samples and 27 IBD samples. The Immunochip, which includes a high density of genotypes at loci containing markers known to be associated with various autoimmune and inflammatory diseases, including CD and UC, was used to genotype the remaining IBD samples. Following QC, imputation was performed using the SHAPEIT and IMPUTE2 software in order to merge the datasets [48, 49]. However, due to the nature of the Immunochip, imputation failed to generate reliable results for sites outside of the densely genotyped regions. Consequently, the eQTL analysis was initially performed independently on the JIA and IBD datasets, and then, overlapping loci significant in either study were pooled for the interaction testing. For JIA, following QC, we analyzed 109 individuals with 5,522,769 variants. For IBD, the available Affymetrix samples were merged with the remaining 27 IBD samples from the Immunochip dataset by selecting overlapping SNPs, which following QC resulted in 54 individuals with 58,788 variants in the vicinity of the 186 immune-related loci, plus the HLA complex, included on the Immunochip. In summary, 27 IBD samples were genotyped on the Affymetrix array, while 27 were typed on the Immunochip, and the remaining 21 IBD samples had expression but not genotype data.

Using the genes from the SVA and SNM adjusted expression data and the separate compiled variants from JIA and IBD, a list of genes and SNPs within 250 kb upstream and downstream of the stop and start coordinates of the gene was generated. eQTL mapping was performed using the linear mixed modelling method in GEMMA [50], which generated a final file of 16,913,152 SNP-gene pairs for JIA samples and 338,005 SNP-gene pairs for IBD samples. Since there are on average close to five candidate genes per SNP, between the two diseases, 263,575 SNP-gene pairs were shared that were analyzed jointly. A common *p* value threshold of *p* < 0.0001 corresponding to an empirical FDR < 5% was chosen, yielding 814 SNP-gene univariate associations. Conditional analysis was underpowered to detect secondary signals consistently, so we simply retained the peak eSNP associations defining 142 eGenes. Since low minor allele frequencies can drive spurious eQTL signatures if the minor homozygotes have outlier gene expression, we checked for an overall relationship between MAF and eQTL significance. None was observed, implying that rare variants are not driving the results in general, but we also examined each of the loci with significant interaction effects manually, identifying a small number of false positives. A notable example is IL10, which had an anomalously high disease-by-interaction (*p*~10^−7^) driven by a large effect size in IBD (beta = 2.7) that turns out to be due to a single outlier, removal of which abrogates any eQTL effect at the locus (also consistent with the blood eQTL browser report [51]).

The eQTL×disease interaction effect which evaluates whether the genotype contribution is the same in JIA and IBD was modeled by combining the imputed rsID genotypes for the lead SNP in either disease into a joint linear model with gene expression as a function of genotype, disease, and genotype-by-disease interaction, assuming the residuals are normally distributed with a mean of zero. A caveat to this analysis is that the lead SNP (i.e., the one with the smallest *p* value) is not necessarily the causal variant, and secondary SNPs in one or other condition may skew the single-site evaluations. Post hoc analyses revealed that secondary eQTLs are evident at three loci reported (*PAM*, *SLC22A5*, and *GBAP1*).

### Adjustments for medication and disease duration

Because the JIA patients in our study were not recruited from a single cohort, therapeutic interventions and duration of disease vary between individuals. Environmental factors include exposure to medications and impact gene expression profiles [52]. In addition, it has previously been shown that gene expression networks are altered over the first 6 months of therapy for JIA patients [53]. To characterize the effects of these covariates, our JIA patients were classified by three non-exclusive categories of medication: known treatment with DMARDs, biologics, and steroids at the time of sample collection, as well as three categories of disease duration prior to sampling: less than 180 days, 180–360 days, and greater than 360 days. Nearly all IBD patients were sampled at diagnosis, so this stratification was only necessary for JIA patients. Medication and time variables were then modeled and removed using SNM, resulting in an adjusted gene expression dataset [42]. The previously described BIT axis analysis was performed again using this adjusted dataset and compared with results from the unadjusted dataset (Additional file 1: Figure S1A). Additional file 1: Figure S1B shows the correlation between unadjusted gene expression and category of disease duration. In addition, the JIA eQTL study was rerun using the adjusted expression dataset. The correlation of betas from the unadjusted and adjusted analyses is depicted in Additional file 1: Figure S2.

Furthermore, we were able to replicate the major trends in gene expression observed in our dataset in a published Affymetrix microarray study of samples from the various subsets of JIA [54]. They studied PBMC gene expression for 29 controls, 30 oligoarticular, 49 polyarticular, and 18 systemic JIA patients all obtained prior to initiation of therapy [54]. As shown in Additional file 1: Figure S3, axes R, B, N, I, and C give very similar results whereas the T cell signature which is mildly reduced in more severe JIA in our data does not differentiate their sample types. Additionally, axis G reverses the sign of effect, as it does upon adjustment for medication usage, reinforcing the conclusion that general cellular metabolic processes are affected by medication. By contrast, Hu et al. [55] report effects of anti-TNF biologic therapy specifically on certain neutrophil-related pathways, a result not recapitulated in our data, likely due to differences in experimental design.

### Colocalization and transcriptional risk score (TRS) analysis

Colocalization analysis was performed using JIA and IBD eQTL data and prior IBD, rheumatoid arthritis, and JIA GWAS study data. The coloc R package uses a Bayesian model to determine posterior probabilities for five hypotheses on whether a shared causal variant is present for two traits [56]. The analysis considered all SNPs associated with IBD (*n* = 232), RA (*n* = 101), or JIA (*n* = 28) as discovered by GWAS, where *n* = 198, 57, 21 and *n* = 198, 83, 20 were present in SNP-gene eQTL datasets for IBD and JIA, respectively. Cross-comparisons between both of the eQTL datasets and each of the GWAS studies’ reported loci was performed, following which select SNP-gene pairs with high probabilities of hypothesis 3 (same locus but different eQTL and GWAS peaks) and 4 (same causal variant driving the signal at the eQTL and GWAS peaks) were plotted using LocusZoom [57] to visualize the region surrounding the variants.

Two independent transcriptional risk scores (TRS) were generated using GWAS results for IBD [58] and RA [59] as a proxy for JIA (since the JIA pool of variants is currently too small). As previously described, TRS sums the *z*-scores of gene expression polarized by the direction of effect of the eQTL relative to the GWAS risk allele [60]. Thus, if the risk genotype is associated with decreased expression, we invert the *z*-score in the summation such that positive TRS represents elevated risk. We only used genotypes that are validated as both eQTL and GWAS by H4 in the coloc analysis, taking the eQTL list from the blood eQTL browser since it has much higher power than the small disease samples. Thirty-nine and 23 genes were included in the IBD and RA TRS, respectively, as listed in Additional file 2: Table S1. ANOVA was performed between groups to establish whether the TRS can be used to predict disease from blood gene expression.

## Results

### Heterogeneity of gene expression within and among disease sub-types

In order to contrast the nature of differential gene expression between three sub-types of JIA and two sub-types of IBD as well as relative to healthy controls, we conducted whole blood gene expression profiling on a combined sample of 202 children with disease onset between the ages of 0.7 and 17. The sample included 43 cases of oligoarticular JIA, 46 of polyarticular JIA, 26 of systemic JIA, 60 of Crohn’s disease, and 15 of ulcerative colitis. RNA-Seq analysis was performed with a median of 19.6 million paired-end 100 bp reads per sample. After normalization and quality control as described in the “Methods” section, a total of 11,614 genes remained for analysis.

**Fig. 1:**
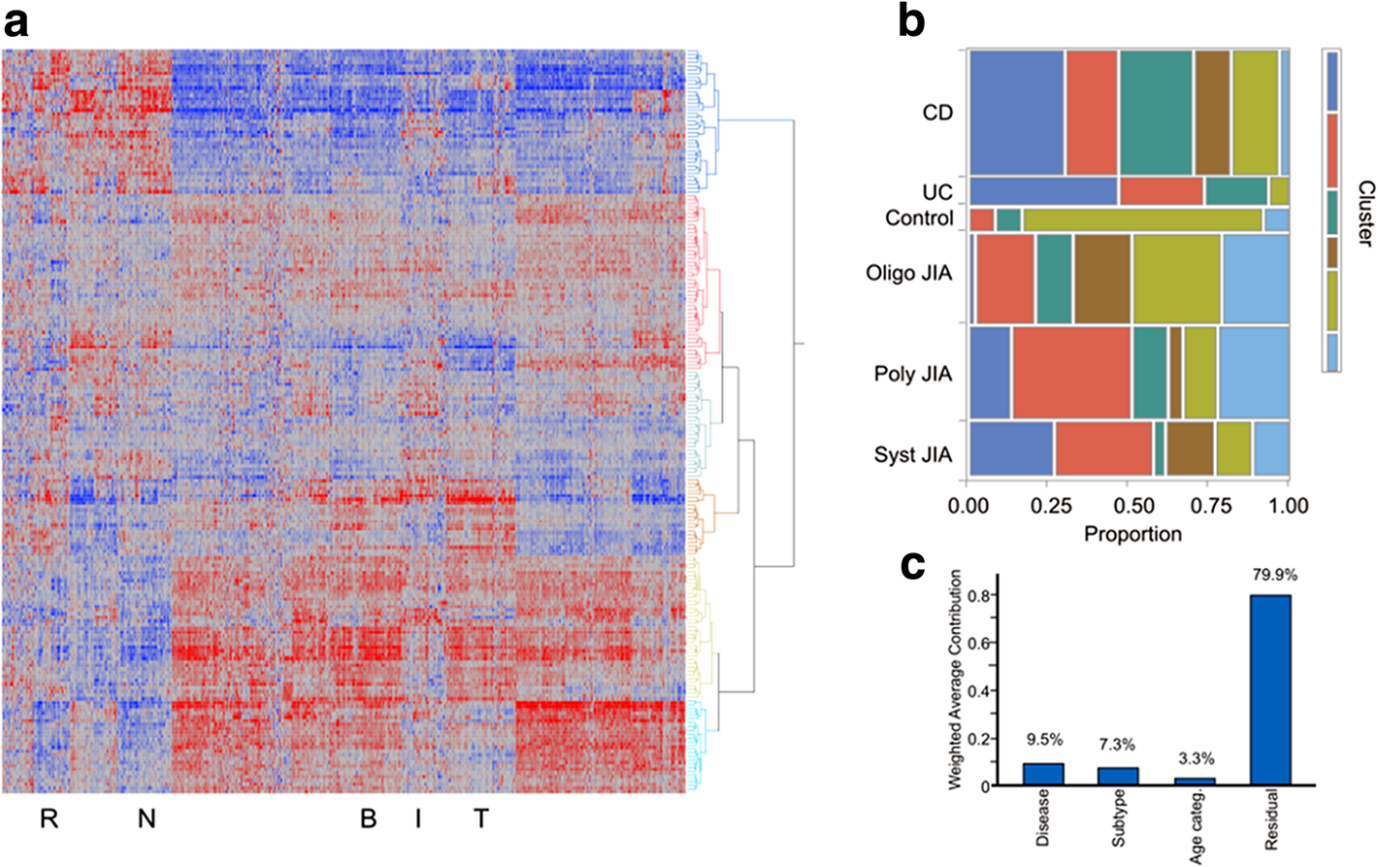
Heterogeneity of gene expression within and among disease sub-types. **a** Two-way hierarchical clustering using Ward’s method of standardized normal (*z*-scores) of transcript abundance of 11,614 genes (columns) in 202 individuals (rows). Six clusters identified to the right group individuals with similar profiles with respect to at least nine clusters of co-expressed genes. Letter beneath the heat map highlight BIT corresponding to genes enriched in reticulocytes (R), neutrophils (N), B cells (B), T cells (T), or for the interferon response (I). **b** Proportion of individuals of each disease sub-type represented in each of the six clusters of individual. For example, 45% of the UC samples are in the dark blue cluster, 30% in the red, 20% in the green, and 5% in the pale green, with none in the brown or light blue. **c** Principal variance component analysis shows the weighted average contribution of disease, sub-type within disease, or age-of-onset before 6 to the first five PC (67%) of the total gene expression variance, with the remainder residual variance unexplained, including individual differences

Previous microarray-based gene expression profiling of JIA has established significant mean differences among disease sub-types, as well as heterogeneity within sub-types [6–9]. A heat map of two-way hierarchical clustering of all genes in all individuals reveals six major clusters of individuals (rows in Fig. 1a) who share co-regulation of at least nine sets of genes (columns). For example, the top cluster labeled in dark blue consists of individuals with generally high innate immunity gene expression and low lymphocyte gene expression, whereas the bottom two clusters labeled in pale blue and green have the opposite profile, though with differences in T cell-related expression. Individuals in each of the six health and disease categories are dispersed throughout the matrix but with highly significant tendencies for enrichment of specific expression clusters in each sub-type, as shown in Fig. 1b. Eighty percent of the healthy controls are in the pale green cluster, which accounts for just one quarter of the oligo-JIA sub-type and less than 15% of each of the others. The two IBD sub-types are more likely to be in the dark blue cluster, as are sJIA cases, consistent with these being more inflammatory conditions, but in each case, the majority of individuals from each disease sub-type are dispersed throughout the other clusters. JIA in general has high membership in the red cluster, while there is an apparent gradient with oligo-JIA more control-like and sJIA more IBD-like. As with other autoimmune diseases, although there are certainly disease-related trends, the overall blood gene expression pattern is dominated by heterogeneity without ambiguous separation by disease type. Figure 1c shows that 9.5% of the gene expression captured by the first five principal components is among disease categories and another 7.3% among the sub-types within JIA and IBD, with a small component also attributable to age-of-onset less than 6.

### Functional characterization of the gradient of differential expression

Contrasts of significant differential expression performed between healthy controls and sub-types of JIA as well as combined IBD and sub-types of JIA confirm the gradient of differential expression between disease groups of different severities. Additional file 2: Table S2 lists the significantly differentially expressed genes at the 5% Benjamini-Hochberg false discovery rate, for each comparison of two disease groups from the six under consideration. In the comparison between healthy controls and oligoarticular JIA, 82 genes were significantly upregulated in healthy controls, and 7 were upregulated in oligoarticular JIA. These numbers are lower than the 136 and 36 differentially expressed genes found in the contrasts between healthy controls and polyarticular JIA, and the 216 and 547 upregulated genes found between healthy controls and sJIA. A similar graded pattern of differentiation was found in comparisons of IBD and JIA. The fewest differentially expressed genes were found in the contrast between IBD and sJIA, with 73 upregulated genes in IBD and 170 upregulated genes in systemic JIA. Between IBD and polyarticular JIA, 934 upregulated IBD genes and 767 upregulated polyarticular genes were discovered, while the biggest differentiation was observed between IBD and oligoarticular JIA, where 2038 upregulated IBD genes and 1751 upregulated oligoarticular genes were discovered. These patterns of differential expression also confirm that of the three JIA sub-types, systemic JIA is the most similar to IBD.

The biological meaning of these differentially expressed genes was investigated through gene ontology and modular analysis. Contrasts between healthy controls and JIA subtypes implied a variety of classes of differential pathway regulation. Overall, all subtypes of JIA showed downregulation of transmembrane signaling and G-protein-coupled receptor activity. However, oligoarticular JIA showed primarily upregulation of protein and phospholipid metabolic processes while polyarticular JIA showed upregulation in secretion, exocytosis, and granulocyte activation, as well as neutrophil activation. Systemic JIA showed an even more strongly significant upregulation of immune pathways, notably general immune response and myeloid activation. In contrast, for the comparisons between IBD and JIA subtypes, all JIA subtypes showed upregulation of nucleic acid processes compared with IBD. Both oligoarticular and polyarticular JIA showed strongly significant downregulation of myeloid, neutrophil, and leukocyte activity compared with IBD, whereas sJIA showed downregulation of general metabolic processes albeit at a much lower significance level.

### Clustering by BTMs and BITs further reveals enriched immune pathways

**Fig. 2:**
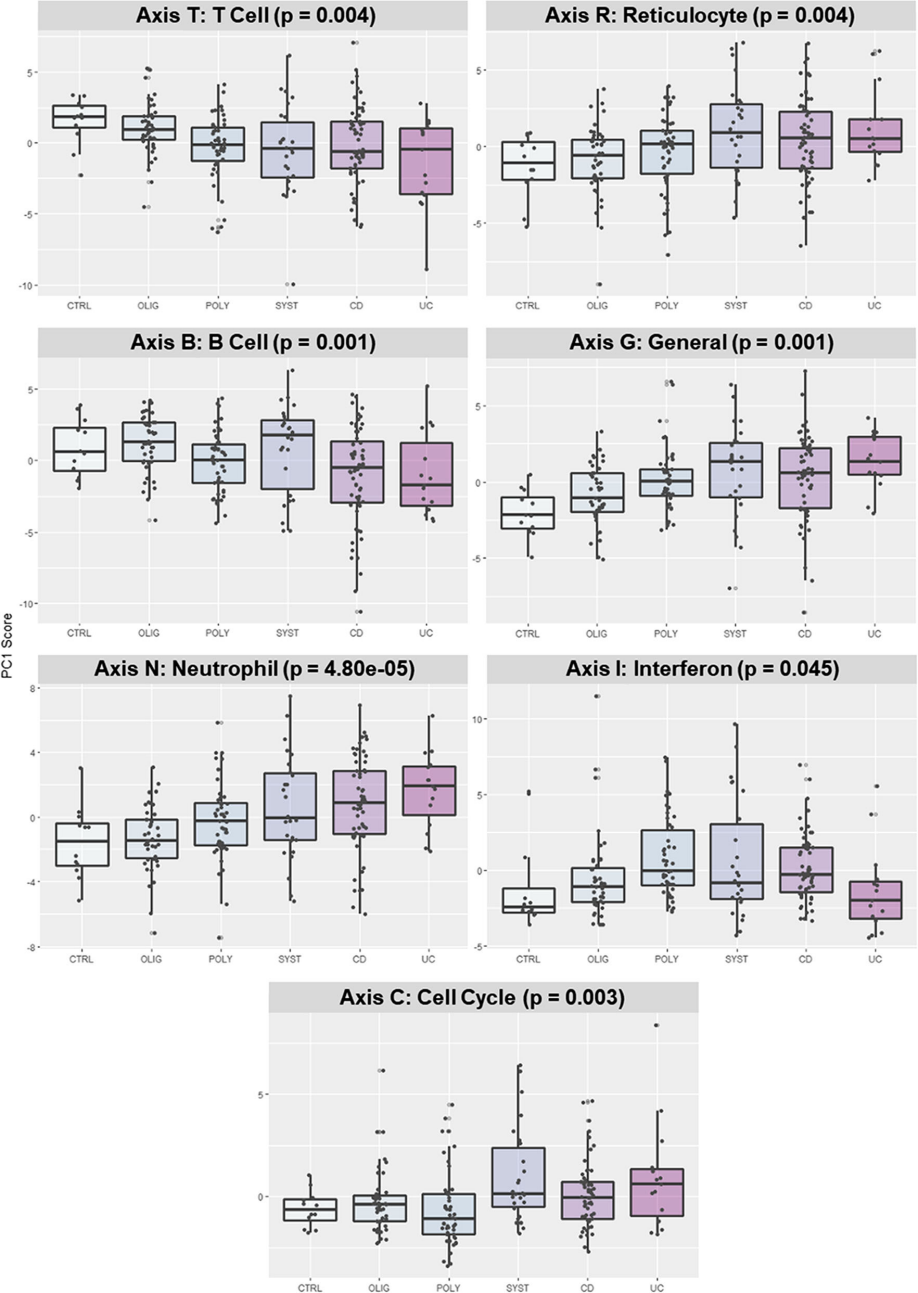
Axes of variation across disease sub-types. Axes of variation defined by the first PC of the Blood Informative Transcripts (BIT) highlight variation in types of immune activity across disease sub-types. Each individual data point represents PC1 score for 10 BIT for the indicated axis, with box and whisker plots showing the median and interquartile range as well as 95% confidence intervals for the sub-types. Indicated *p* values are from one-way ANOVA contrasting the six sub-types of sample

Decades of blood gene expression analysis have highlighted the existence of modules of co-expressed genes that reflect a combination of joint regulation within cell types and variable abundance of the major leukocyte classes [61]. Seven highly conserved axes of blood variation [44] are composed of genes broadly capturing immune activity related to T and B cells, reticulocytes and neutrophils, interferon response, general biosynthesis, and the cell cycle. Figure 2 shows clear trend expression along these axes correlating with disease sub-type, each panel indicating the level of activation in each immune component in, from left to right, healthy control, oligoarticular JIA, polyarticular JIA, systemic JIA, Crohn’s disease, and ulcerative colitis. Axis T, representing T cell expression, and axis B, representing B cell expression, show a trend of decreasing PC1 values correlating with severity of disease, suggesting downregulation of adaptive immunity in systemic JIA, CD, and UC. In contrast, axis R, representing reticulocytes, and axis N, representing neutrophils, show trends of increasing PC1 values with disease severity that indicates upregulation of the innate immune system in systemic JIA, CD, and UC. Axis I represents interferon-responsive gene expression and has a more parabolic trend, being elevated in polyarticular and systemic JIA and Crohn’s disease, but not ulcerative colitis, reflecting the interferon response’s dual roles in both adaptive and innate immunity. Axes G and C represent general and cell cycle expression, and show trends of higher PC1 values in inflammatory bowel disease and systemic JIA. Despite sample sizes of around 30 patients in each group, ANOVA indicates that the differences are significant in each case.

**Fig. 3:**
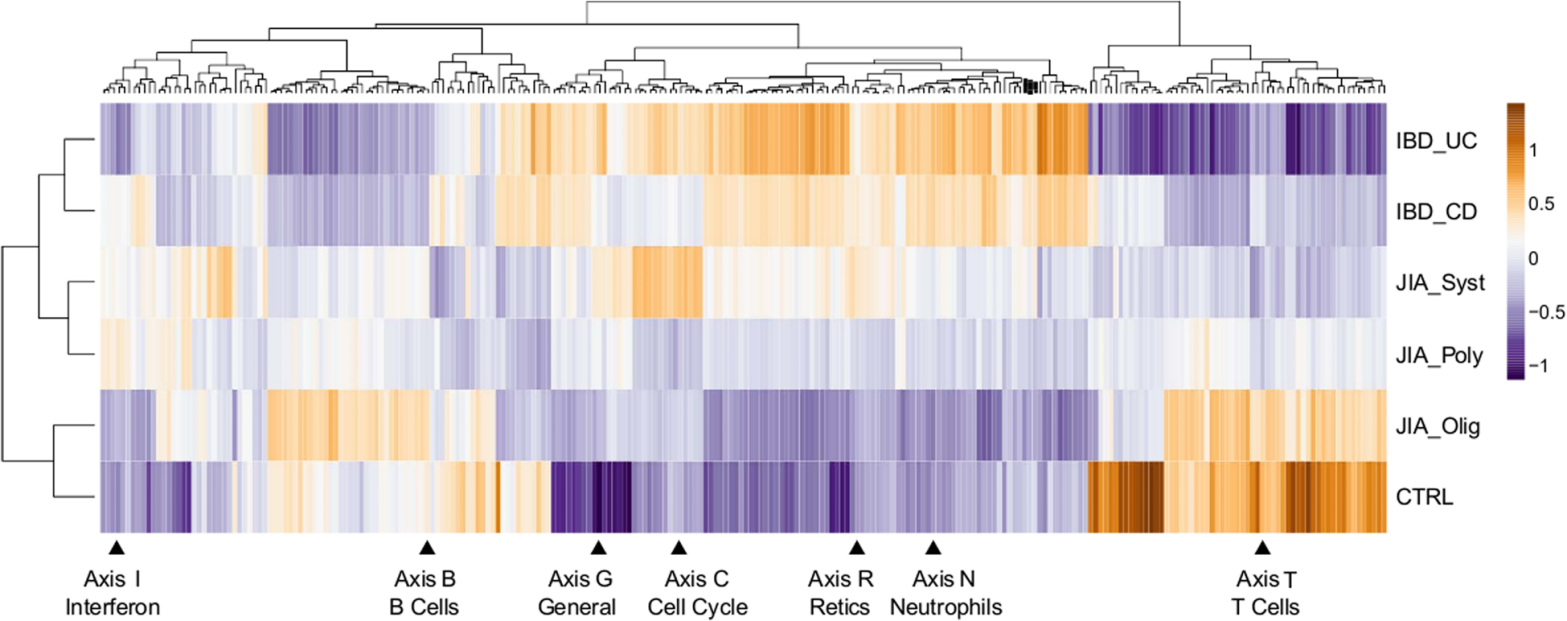
Blood Transcript Modules. Hierarchical clustering of blood transcription modules across disease sub-types. The heat map shows the mean PC1 scores for 247 BTM identified in [45], as well seven BIT axes. Note how the BTM form ~ 10 clusters, seven of which co-cluster with one orthogonally determined axis. See Additional file 2: Table S3 for a complete listing of BTM scores in each disease sub-type

These disease-specific trends are confirmed by hierarchical clustering of 247 Blood Transcript Modules (BTMs) [45] in Fig. 3, tabulated in Additional file 2: Table S3, further supporting the gradient of disrupted gene expression based on disease severity. Healthy controls and oligoarticular JIA show largely similar expression, except for apparent elevation of NK cell gene expression in controls. IBD most resembles sJIA, although with some key differences. Myeloid gene expression tends to be elevated in IBD and lymphoid gene expression suppressed, with JIA intermediate. In addition, ulcerative colitis appears to have a specific deficit in NK cell-biased gene expression, sJIA has a unique signature including inositol metabolism, and JIA in general shows reduced mitochondrial gene activity.

### Transcriptional risk scores differentiate healthy controls, JIA, and IBD

**Fig. 4:**
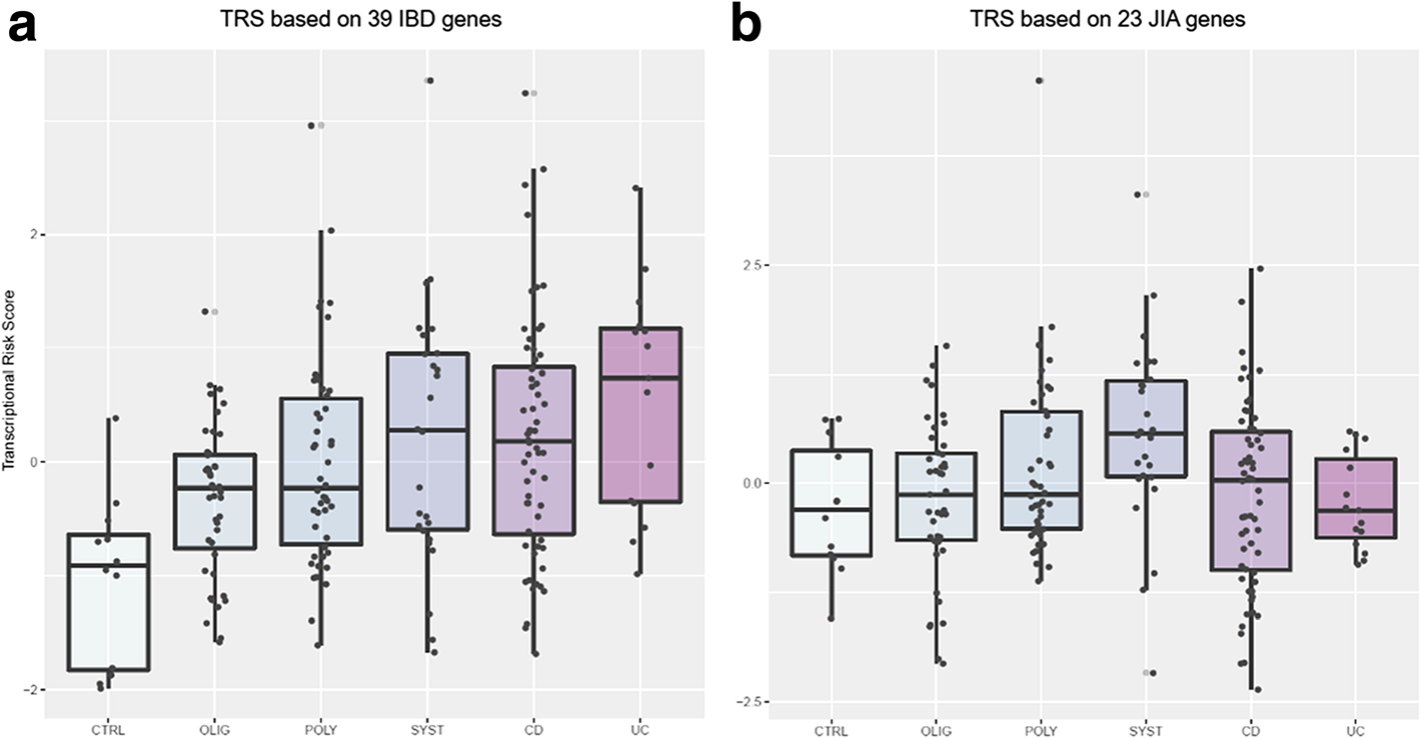
Transcriptional risk scores associate with disease status. **a** IBD-TRS scores within disease sub-types for 39 genes associated with IBD in [58]. Gene expression values for each selected gene were transformed into *z*-scores, polarized relative to risk according to whether the eQTL activity of the risk allele discovered by GWAS increases or decreases transcript abundance, and summed to generate the TRS as in [60]. **b** New RA-TRS based on 23 genes associated with RA by GWAS [59]

We recently proposed the notion of a transcriptional risk score (TRS), which is analogous to a cumulative burden of genotypic risk, but evaluates cumulative burden of risk due to elevated or suppressed gene expression relevant to disease [60, 62]. By just focusing on genes with shared eQTL and GWAS associations, the analysis is restricted to genes most likely to have a causal role in pathology, whether because the risk allele directly promotes disease or fails to provide sufficient protection. A TRS based on eQTL detected in blood but with gene expression measured in ileum was highly predictive of Crohn’s disease progression, whereas a corresponding genetic risk score was not. Figure 4 shows similarly that the 39-gene IBD TRS measured in peripheral blood provides significant discrimination of cases and controls (difference in standard deviation units of TRS; ∆s.d. = 1.10, *p* = 0.0003); notably, sJIA is elevated to the same degree as both CD and UC. By contrast, oligoarticular JIA and polyarticular JIA have intermediate TRS that are nevertheless significantly greater than healthy controls (∆s.d. = 1.04, *p* = 0.0031). For comparison, a TRS based on genes that are likely to be causal in driving the signal at 23 genome-wide significant associations for RA does not discriminate between healthy controls and IBD as a group (∆s.d. = 0.11, *p* = 0.63) but does trend toward discrimination of JIA as a category (∆s.d. = 0.42, *p* = 0.09). This RA TRS is mostly enhanced in sJIA (∆s.d. = 0.86, *p* = 0.008 relative to healthy controls), suggesting that it is capturing the effects of inflammatory gene contributions to this most severe form of JIA.

### Evaluation of disease specificity of eQTL

We next addressed the degree of sharing of the local genetic control of gene expression in the two classes of disease (namely JIA and IBD) by performing comparative eQTL analysis. Whole genome genotypes were ascertained on the Immunochip (CD and UC samples) or the Affymetrix Axiom Biobank array (see the “Methods” section). As far as possible, SNPs were imputed onto the 1000 Genomes reference, allowing cross-comparison of the disease subsets, noting that this was not possible for loci not included on the Immunochip. Since genotypes were generated on different platforms, the eQTL assessment was first performed independently for the two broad disease classes, after which significant effects were evaluated jointly. Here we only consider genes located within the vicinity of the Immunochip loci.

**Fig. 5:**
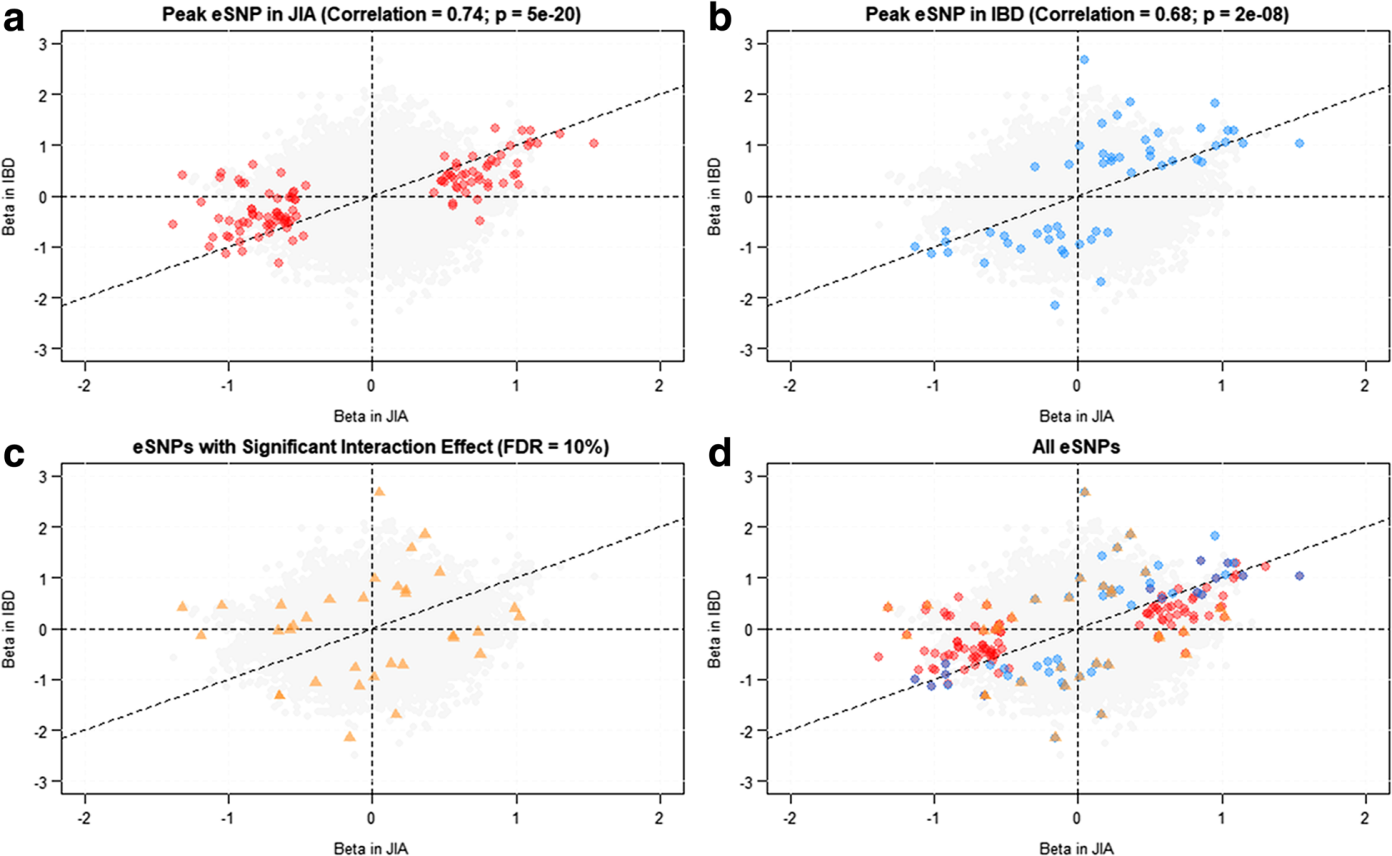
Comparison of peripheral blood eQTL effects between JIA and IBD. Effect sizes of peak eSNPs by disease. **a** Correlation of beta effect sizes between IBD and JIA for the 107 peak independent eSNPs discovered in the JIA sample. **b** Correlation of beta effect sizes between IBD and JIA for the 52 top eSNPs identified in JIA. **c** Thirty-four eSNPs with a significant interaction effect between disease and genotype when evaluated jointly. **d** Overlay of all eSNPs

For JIA, 107 independent eSNPs were identified within 500 kb of a transcript at an FDR of 5% (approximate *p* < 10^−4^), and for IBD, which had a smaller sample size, 52 independent eSNPs were identified. These are listed in Additional file 2: Table S4. Twelve of the loci overlap between the two diseases, but failure to detect an eQTL in one condition does not necessarily imply absence of the effect, since the small sample size results in relatively low power. Overall, the correlation in effect sizes is high, ~ 0.7 (*p* = 5 × 10^−20^ in JIA; *p* = 2 × 10^−8^ in IBD), which is remarkable given the small sample sizes, and strongly implies that most eQTL effects in whole blood are consistent across the diseases. Nevertheless, the plots in Fig. 5 depicting the estimated eQTL effect sizes in IBD relative to JIA provide some support for disease-biased effects in so far as the eQTL discovered in JIA (red points, panel a) tend to have larger effects on JIA (beta values) than those observed in IBD and hence lie between the diagonal and the *x*-axis. Conversely, the eQTL discovered in IBD (blue points, panel b) tend to have larger effects on IBD than those observed in JIA and hence lie between the diagonal and the *y*-axis. This result is biased by winner’s curse, the tendency to over-estimate effect sizes upon discovery, so we also evaluated all associations jointly in order to also identify interaction effects. At an FDR of 10%, 34 of the 147 independent eQTL, highlighted in panel , show nominally significant interaction effects (*p* < 0.02), implying different effect sizes in the two broad classes of disease. Example box plots of genotypic effects on transcript abundance across the two disease classes are provided in Additional file 1: Figure S4. These genotype-by-disease interaction effects remain significant after accounting for ancestry (see Additional file 1: Figure S5).

As expected, many of the detected eQTLs affect expression of genes in the vicinity of established GWAS hits for autoimmune disease. Table 1 lists 25 lead eSNPs that regulate expression in cis of 22 target genes that are listed on ImmunoBase as potential causal genes for IBD or arthritis (JIA or RA). Half of these associations are with IBD only, but this bias may simply reflect increased power of the IBD GWAS to date. Several of the SNPs show evidence of disease-specific or disease-biased effects. Naively, we might expect the eQTL to be seen only in the disease(s) for which the association with disease is seen, as this would be consistent with allele-specific expression driving pathology. Three cases (*ARPC2*, *CPTP* for IBD, and the secondary eQTL in *PAM* for JIA) fit the expected pattern, but three others have the counter-intuitive relationship where the eQTL is observed in one disease but the established GWAS association is with the opposite disease (*PRDX6* and *ADAM1A* for RA, the secondary eQTL in *GBAP1* for CD). Three more cases (*SLC22A5*, *CD226*, and *RNASET2*) have possibly disease-biased eQTL effects where the eQTL is absent from or much less in one disease, although the interaction effect is only significant in one of these cases. Despite the small sample, there is not an intuitive pattern to the relationship between disease-biased regulation of gene expression and association with disease.

**Table 1:** GWAS eQTL

Gene	rsID	IBD β	IBD *p* val	JIA β	JIA *p* val	IBD-GWAS	ATH-GWAS	Interact *p*
*ARPC2*	rs13429408	0.82	6.60E−05	0.18	0.22	CD, UC	–	0.01
*CPTP*	rs11809901	− 1.08	9.80E−05	− 0.12	0.69	CD, UC	–	0.04
*PAM*	rs2431321	1.04	3.80E−09	1.15	2.10E−23	–	RA	0.48
*PAM*	rs32677	0.21	0.3	0.94	5.30E−15	–	RA	9.60E−05
*C5*	rs1468673	0.39	0.02	0.74	3.10E−07	–	RA	0.34
*PRDX6*	rs4279882	1.84	3.80E−05	0.36	0.05	–	RA	0.001
*ADAM1A*	rs11066027	1.22	2.40E−05	0.61	5.30E−03	–	JIA, RA	0.09
*RNASET2*	rs385863	− 0.68	1.30E−04	− 1.05	1.40E−14	CD, UC	RA	0.3
*GSDMB*	rs11078926	− 0.51	5.90E−03	− 0.56	9.90E−07	CD, UC	RA	0.87
*SLC22A5*	rs11739135	0.09	0.6	− 0.8	9.80E−10	CD, UC	JIA	4.00E−05
*SLC22A5*	rs11950562	− 0.53	8.00E−04	− 0.86	6.10E−14	CD, UC	JIA	0.07
*ORMDL3*	rs1565923	1.11	8.80E−07	0.47	6.20E−04	CD, UC	RA	0.01
*ICAM4*	rs3093029	1.22	4.80E−04	1.3	2.90E−08	CD, UC	JIA	0.69
*RMI2*	rs11644184	− 0.58	7.60E−04	− 0.7	3.00E−07	CD, UC	JIA	0.54
*PLTP*	rs7275164	− 0.56	2.10E−04	− 0.71	7.00E−07	CD, UC	RA	0.58
*CD226*	rs12969613	0.63	2.20E−07	0.18	0.15	CD, UC	RA	0.11
*NOD2*	rs1981760	1.28	2.70E−08	1.05	2.30E−16	CD	–	0.23
*GBAP1*	rs914615	0.6	3.20E−04	0.8	7.80E−10	CD	–	0.62
*GBAP1*	rs3814319	0.16	0.33	0.7	1.20E−06	CD	–	0.05
*KSR1*	rs2945378	− 0.48	6.20E−03	− 0.6	4.40E−07	CD	–	0.52
*SULT1A1*	rs7191548	− 0.49	6.50E−03	− 0.61	5.30E−07	CD, UC	–	0.93
*PNKD*	rs13430006	0.34	0.14	0.57	6.80E−07	CD, UC	–	0.41
*NLRP2*	rs12975582	0.56	0.01	0.8	1.20E−06	CD, UC	–	0.43
*SLC11A1*	rs78846874	− 0.35	0.36	− 0.83	3.90E−06	CD, UC	–	0.22
*LGALS9*	rs1984547	− 0.88	2.40E−05	− 0.55	4.10E−05	CD, UC	–	0.16

**Fig. 6:**
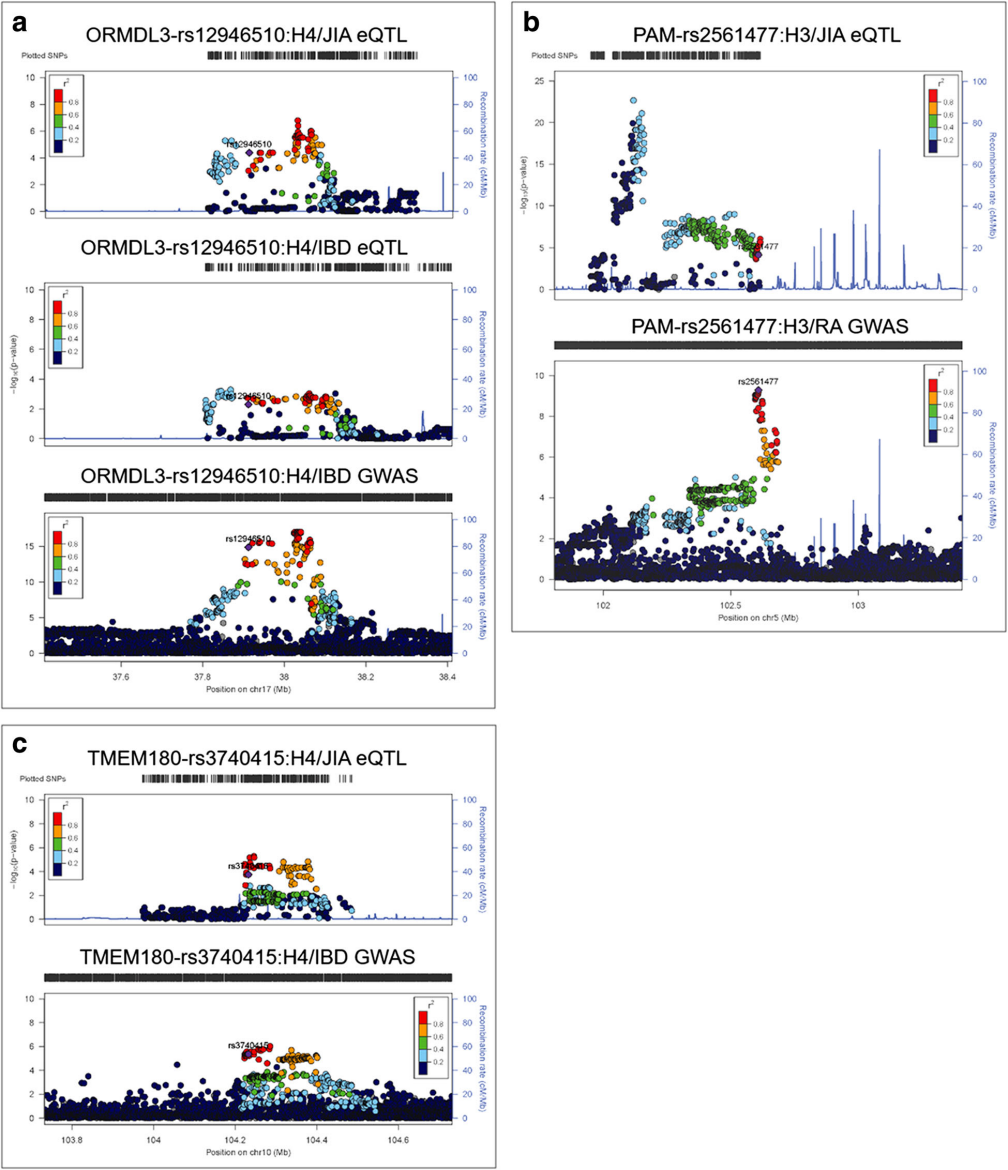
Colocalization of eQTL and GWAS signatures. LocusZoom plots show the univariate SNP-wise association statistics for each genotyped SNP either with the abundance of the indicated trasncript (eQTL effects) or from the GWAS for IBD or RA. Color coding indicates the *r*^2^ measure of linkage disequilibrium of each SNP with the relevant peak GWAS SNP. **a** rs12946510 is most likely a shared causal variant for *ORMDL3* gene expression in both IBD and JIA, as well as in the IBD GWAS. However, a likely secondary signal in the light blue region is not associated with IBD. **b** rs2561477 is the peak causal variant in RA but clearly does not colocalize with the peak eQTL for JIA. **c** rs3740415 is most likely a shared causal variant for expression of *TMEM180* and in the IBD GWAS despite an extensive LD block at the locus (though it does not meet the strict GWAS threshold)

One reason for divergent effect sizes may be that different causal variants in variable degrees of linkage disequilibrium could be responsible for the differential expression in the two disease sub-types. To investigate this, we performed colocalization analysis using coloc [56] to visualize the locus-wide SNP effects across all loci reported in IBD, RA, and JIA GWAS and present in our SNP-gene datasets for IBD or JIA and compared these with the distribution of GWAS summary statistics. Coloc assigns a posterior probability that the same SNP is responsible for both an eQTL effect and the disease association (H4) or that different SNPs are responsible for the two effects (H3). Since the power of this mode of analysis is limited when sample sizes are small, we identified cases from either disease with relatively strong H3 or H4 posterior probabilities and plotted representative examples in Fig. 6. The full results are summarized in Additional file 2: Table S5.

Figure 6a shows results for association of rs12946510 with IBD from GWAS (bottom panel) and the eQTL profiles for the JIA (top panel) and IBD (middle panel) gene expression. Although coloc calls both cases as H4, the correspondence of SNP profiles in high LD with the lead SNP is more notable in JIA. The light blue SNPs suggest a second, independent, eQTL which does not produce a GWAS signal. Hence, the gene expression difference may be mediated by two different SNPs, possibly with different effect sizes in the two diseases, only one of which appears to contribute strongly to disease risk. Figure 6b shows a clear H3 case in JIA where the eQTL effect on expression of *PAM* appears to be mediated by a cluster of variants to the left of the lead GWAS cluster. Figure 6c shows a classical H4 where the fine mapping supports a single causal locus for both the gene expression and disease, although the precise identity of the causal variant is impossible to ascertain from the statistical data alone owing to the extensive block of variants in high LD.

## Discussion

### Disease-specific associations with autoimmune disease

There are multiple technical reasons why GWAS may fail to detect associations that are shared across multiple autoimmune diseases. These include differences in sample size and clinical heterogeneity, and with respect to eQTL analysis, differences in expression profiling platform, statistical methodology, and effects of pharmacological interventions could all obscure associations. However, it is also clear that the genetic correlation across diseases is significantly less than one, establishing the expectation that some effects must be disease-specific [63]. The most appropriate framework for detecting such effects is evaluation of the significance of genotype-by-disease interaction terms, which motivated the current study.

The core result of the comparative eQTL component of this study is that the majority of genetic influences on transcript abundance measured in whole blood are consistent across IBD and JIA. A major caveat to this conclusion is that immune cell sub-type specific effects will often go undetected in both whole blood and PBMC studies [14, 18]. It is though important to note that while neutrophils, lymphocytes, macrophages, and monocytes certainly do have unique and disease-relevant eQTL, comparative studies also confirm that over three quarters of eQTL are shared by the majority of immune cells [64, 65].

Just as importantly, equivalence of genetic influences on gene expression does not necessarily mean equivalence of genetic influences on disease susceptibility. Among the shared eQTL, some numbers are still likely to be specific to CD, UC, JIA, or other conditions by virtue of other influences. These may include disease-specific contributions of the critical cell type, environmental differences (for example, microbial infection of the gut may elevate or suppress expression of the gene to a degree that renders the eQTL meaningful or irrelevant), or interactions with the genetic background (for example, elevated expression of a gene may only matter in the context of other genetic risk factors). Although there is little evidence that two-locus genotype-by-genotype interactions contribute meaningfully to heritability [66], renewed interest in influences of overall genetic risk on the impact of specific genotypes makes sense given the context of gene expression heterogeneity [67].

Our analyses do provide evidence that as many as 20% of eQTL effects in peripheral blood may at least show disease-specific biases. Such differences in effect sizes are likely to trace to differences in the expression of transcription factors and epigenetic modifications between diseases and/or to differences in the relative abundance of contributing cell types. Methods exist for deconvoluting effects of cell-type abundance [68], but they are low resolution and in our opinion unreliable when applied to sample sizes of the order of 100; next-generation studies incorporating single-cell RNA-Seq will be much more informative.

The relationship between disease-specific eQTL and GWAS association at the same locus is less straightforward than might be expected under the assumption that the effect of a polymorphism on disease is mediated through its effect on transcription of the associated gene. It is not immediately clear why an eQTL may only be detected in one disease while the GWAS association is in another disease, yet multiple instances are found in our data. This observation adds to a growing body of data questioning whether detected eQTL effects explain causal associations. Two fine mapping studies of IBD published in 2017 [69, 70] both found less than 30% identity between mapped eQTL and GWAS causal intervals, one suggesting that there is more significant overlap with methylation QTL and both arguing that the relevant effects may be specific to particular cell types or activation conditions, including immune activity at the sight of the pathology. Additionally, we described a meaningful number of “incoherent” associations, where mean differential expression between cases and controls is in the opposite direction to that predicted by the effect of the risk allele on gene expression [60]. Such results highlight the need for a combination of fine structure mapping of causal variants and detailed mechanistic studies of immune cell-type contributions if we are to fully understand how segregating polymorphisms contribute to disease susceptibility and progression.

### Disease- and sub-type-specific gene expression

Numerous other studies have described gene expression profiles in a variety of inflammatory autoimmune diseases, but we are aware of just a single side-by-side comparison of two or more diseases on the same platform [65]. Straightforward cluster analysis shows that both IBD and JIA subjects tend to differ from healthy controls, but they have overall transcriptome profiles that may belong to a half dozen types. Blood Transcript Module and BIT axis analyses, both based on comprehensive analysis of existing whole blood gene expression datasets, confirm that these types broadly reflect differences in gene activity in the major immune sub-types, partly reflecting cell abundance, but also innate states of activity of biosynthetic, cell cycle, and cytokine signaling. Immunoprofiling by flow cytometry has established that individuals have baseline profiles, or omic personalities [71], to which they return after immunological perturbation but which are also influenced by such environmental factors as child-rearing [72]. Sub-type-specific blood gene expression should be seen in light of this immunological elasticity, as the heterogeneity among subjects may be more meaningful for disease risk than individual eQTL effects.

Juvenile idiopathic arthritis is the most prevalent childhood rheumatic disease, encompassing multiple physically, immunologically, and genetically different sub-types of disease. Although diagnosis and classification is based upon largely clinical criteria, the genetic complexity of JIA has been well documented [27, 28]. While the oligoarticular and polyarticular sub-types demonstrate activation of adaptive immunity, systemic JIA appears to be mediated more heavily through innate immunity, and profiles of immune cell activity between sub-types differ [73–75]. These findings at the gene expression level are consistent with emerging GWAS results suggesting that systemic JIA is etiologically a quite different disease. It is particularly noteworthy that both of the transcriptional risk scores we document show that systemic JIA is divergent from the articular forms, being close to the IBD profiles for the IBD-TRS, and uniquely elevated for the RA-TRS.

In this study, we performed cross-sub-type and disease comparisons of gene expression and eQTLs to characterize the similarities and differences between the forms of JIA. Differential gene expression analysis revealed a gradient of order among the JIA sub-types and IBD, from healthy controls, to oligoarticular, polyarticular, and systemic JIA, to Crohn’s disease and ulcerative colitis. Numbers of differentially expressed genes, gene ontology pathway types, and significance levels agree with this pattern of ordering. Consistent with previous research, oligoarticular and polyarticular JIA exhibits a trend of activated T cell gene expression relative to systemic JIA [17–20, 23]. As a group, JIA also demonstrates increased expression of B cell-related genes. There is also an ordered increase in neutrophil gene expression from oligoarticular to systemic JIA, which concurs with systemic JIA being closely tied with innate immunity. In addition, the elevation of oligoarticular and polyarticular JIA over controls points to involvement of neutrophils in these sub-types as well, which has been previously suggested [5]. Taken as a whole, these findings suggest that JIA sub-types are mediated through a complex relationship between adaptive and innate immunity, and neither disease can be fully characterized by simply one or the other.

### Limitations

This study has three major limitations. Firstly, since the subjects were not a part of any single-cohort study, they were treated with different medications or had samples taken at later time points after diagnosis. The sample size, though larger than many published studies, is still too small to partition the effects of plausible technical covariates or of environmental mediators of gene expression such as those described by Favé et al. and Idaghdour et al. [52, 76]. The results of the covariate-adjustment analyses presented in Additional file 1: Figures S1 and S2 suggest that the effects on our dataset are minimal compared with the consistent effect of disease subtype, but therapeutic effects should still be considered in interpretations of our findings. Secondly, whole blood samples were utilized to measure gene expression. Because whole blood is composed of multiple cell types, there will inherently be some mixture and dilution of gene signatures. Although it is well established that whole blood expression profiles are capable of illuminating aspects of autoimmune pathology, immune cell sub-type analyses will have higher resolution [18]. Single-cell RNA-Seq has great potential both to trace general features of peripheral blood gene expression to specific cell types and to foster accurate eQTL analysis at the sub-type level. Thirdly, we describe just a cross-sectional snap shot of the transcriptome of each subject, whereas longitudinal profiling has the promise of correlating personalized transcriptional shifts to clinical response [77].

## Conclusions

Gene expression and genotyping data can help to categorize sub-types of JIA and IBD beyond just clinical features. The gradient of gene expression from healthy controls to oligoarticular, polyarticular, and systemic JIA to IBD reflects a complex interplay between adaptive and innate immunity responsible for differentiation between JIA sub-types. Individuals have sub-type-specific probabilities of having one of a small number of global gene expression profiles. Since the majority of eQTL appear to have similar effect sizes across disease sub-types, disease-specific eQTL effects only explain a small fraction of disease-specific genetic influences on disease. Considerably more fine mapping and functional analysis will be required before personalized therapeutic interventions for patients with distinct forms of JIA or IBD become commonplace.

## Additional files

Additional file 1: **Figure S1.** Effects of medication and sample time on gene expression. **Figure S2.** Correlation of betas in non-adjusted and medication-adjusted SNPs. **Figure S3.** Replication of gene expression trends in the Hinze et al. dataset. **Figure S4.** Examples of disease-specific eQTL. Figure S5. Interaction effects with addition of ethnicity. (PDF 630 kb)

Additional file 2: **Table S1.** List of genes included in transcriptional risk scores. **Table S2.** List of differentially expressed genes. **Table S3.** BTM across disease sub-types. **Table S4.** List of disease-by-eQTL interactions. Table S5. Colocalization analysis. (XLSX 329 kb)

## Abbreviations

BIT: Blood Informative Transcript
BTM: Blood Transcription Module
CD: Crohn’s disease
eQTL: Expression quantitative trait locus
GWAS: Genome-wide association study
HLA: Human leukocyte antigen
IBD: Inflammatory bowel disease
JIA: Juvenile idiopathic arthritis
mQTL: Methylation quantitative trait locus
RA: Rheumatoid arthritis
TRS: Transcriptional risk score
UC: Ulcerative colitis

## References

[1] Gutierrez-Arcelus M, Rich SS, Raychaudhuri S (2016). Autoimmune diseases—connecting risk alleles with molecular traits of the immune system. Nat Rev Genet.

[2] McGovern DP, Kugathasan S, Cho JH (2015). Genetics of inflammatory bowel diseases. Gastroenterology.

[3] Nédélec Y, Sanz J, Baharian G, Szpiech ZA, Pacis A, Dumaine A (2016). Genetic ancestry and natural selection drive population differences in immune responses to pathogens. Cell.

[4] Ye CJ, Feng T, Kwon HK, Raj T, Wilson MT, Asinovski N (2014). Intersection of population variation and autoimmunity genetics in human T cell activation. Science.

[5] Jarvis JN, Petty HR, Tang Y, Frank MB, Tessier PA, Dozmorov I (2006). Evidence for chronic, peripheral activation of neutrophils in polyarticular juvenile rheumatoid arthritis. Arthritis Res Ther.

[6] Ogilvie EM, Khan A, Hubank M, Kellam P, Woo P (2007). Specific gene expression profiles in systemic juvenile idiopathic arthritis. Arthritis Rheumatol..

[7] Barnes MG, Grom AA, Thompson SD, Griffin TA, Pavlidis P, Itert L (2009). Sub-type-specific peripheral blood gene expression profiles in recent-onset juvenile idiopathic arthritis. Arthritis Rheumatol..

[8] Jiang K, Sawle AD, Frank MB, Chen Y, Wallace CA, Jarvis JN (2014). Whole blood gene expression profiling predicts therapeutic response at six months in patients with polyarticular juvenile idiopathic arthritis. Arthritis Rheumatol..

[9] Prahalad S, Zeft AS, Pimentel R, Clifford B, McNally B, Mineau GP (2010). Quantification of the familial contribution to juvenile idiopathic arthritis. Arthritis Rheumatol..

[10] Ravelli A, Martini A (2007). Juvenile idiopathic arthritis. Lancet.

[11] Macaubas C, Nguyen K, Milojevic D, Park JL, Mellins ED (2009). Oligoarticular and polarticular JIA: epidemiology and pathogenesis. Nat Rev Rheumatol.

[12] Mellins ED, Macaubas C, Grom AA (2011). Pathogenesis of systemic juvenile idiopathic arthritis: some answers, more questions. Nat Rev Rheumatol.

[13] Singh-Grewal D, Schneider R, Bayer N, Feldman BM (2006). Predictors of disease course and remission in systemic juvenile idiopathic arthritis: significance of early clinical and laboratory features. Arthritis Rheumatol..

[14] Cui A, Quon G, Rosenberg AM, Yeung RSM, Morris Q, BBOP Study Consortium. Gene expression deconvolution for uncovering molecular signatures in response to therapy in juvenile idiopathic arthritis. PLoS One 2016;11:e0156055. 10.1371/journal.pone.0156055 PMC4887077 27244050

[15] Jarvis JN, Frank MB (2010). Functional genomics and rheumatoid arthritis: where have we been and where should we go?. Genome Med.

[16] Wouters CH, Ceuppens JL, Stevens EA (2002). Different circulating lymphocyte profiles in patients with different sub-types of juvenile idiopathic arthritis. Clin Exp Rheumatol.

[17] Griffin TA, Barnes MG, Ilowite NT, Olson JC, Sherry DD, Gottlieb BS (2009). Gene expression signatures in polyarticular juvenile idiopathic arthritis demonstrate disease heterogeneity and offer a molecular classification of disease subsets. Arthritis Rheum.

[18] Wong L, Jiang K, Chen Y, Hennon T, Holmes L, Wallace CA, Jarvis JN (2016). Limits of peripheral blood mononuclear cells for gene expression-based biomarkers in juvenile idiopathic arthritis. Sci Rep.

[19] Barnes MG, Grom AA, Thompson SD, Griffin TA, Luyrink LK, Colbert RA, Glass DN (2010). Biologic similarities based on age at onset in oligoarticular and polyarticular sub-types of juvenile idiopathic arthritis. Arthritis Rheumatol..

[20] Macaubas C, Nguyen K, Deshpande C, Phillips C, Peck A, Lee T (2010). Distribution of circulating cells in systemic juvenile idiopathic arthritis across disease activity states. Clin Immunol.

[21] ImmunoBase. Juvenile Diabetes Research Foundation/Wellcome Trust Diabetes and Inflammation Laboratory 2018. https://www.immunobase.org. Accessed 5 Feb 2018.

[22] Prahalad S, O-Brien E, Fraser AM, Kerber RA, Mineau GP, Pratt D (2004). Familial aggregation of juvenile idiopathic arthritis. Arthritis Rheumatol..

[23] Hinks A, Bowes J, Cobb J, Ainsworth HC, Marion MC, Comeau ME (2017). Fine-mapping the MHC locus in juvenile idiopathic arthritis (JIA) reveals genetic heterogeneity corresponding to distinct adult inflammatory arthritic diseases. Ann Rheum Dis.

[24] Hersh AO, Prahalad S (2015). Immunogenetics of juvenile idiopathic arthritis: a comprehensive review. J Autoimmun.

[25] Thompson SD, Sudman M, Ramos PS, Marion MC, Ryan M, Tsoras M (2010). The susceptibility loci juvenile idiopathic arthritis shares with other autoimmune diseases extend to PTPN2, COG6, and ANGPT1. Arthritis Rheumatol..

[26] Thompson SD, Marion MC, Sudman M, Ryan M, Tsoras M, Howard TD (2012). Genome-wide association analysis of juvenile idiopathic arthritis identifies a new susceptibility locus at chromosomal region 3q13. Arthritis Rheumatol.

[27] Hinks A, Cobb J, Marion MC, Prahalad S, Sudman M, Bowes J (2013). Dense genotyping of immune-related disease regions identifies 14 new susceptibility loci for juvenile idiopathic arthritis. Nat Genet.

[28] McIntosh LA, Marion MC, Sudman M, Comeau ME, Becker ML, Bohnsack JF (2017). Genome-wide association meta-analysis reveals novel juvenile idiopathic arthritis susceptibility loci. Arthritis Rheumatol..

[29] Stock CJ, Ogilvie EM, Samuel JM, Fife M, Lewis CM, Woo P (2008). Comprehensive association study of genetic variants in the IL-1 gene family in systemic juvenile idiopathic arthritis. Genes Immun.

[30] Fife MS, Gutierrez A, Ogilvie EM, Stock CJ, Samuel JM, Thomson W (2006). Novel IL10 gene family associations with systemic juvenile idiopathic arthritis. Arthritis Res Ther..

[31] Ombrello MJ, Arthur VL, Remmers EF, Hinks A, Tachmazidou I, Grom AA (2017). Genetic architecture distinguishes systemic juvenile idiopathic arthritis from other forms of juvenile idiopathic arthritis: clinical and therapeutic implications. Ann Rheum Dis.

[32] Di Narzo AF, Peters LA, Argmann C, Stojmirovic A, Perrigoue J, Li K (2016). Blood and intestine eQTLs from an anti-TNF-resistant Crohn’s disease cohort inform IBD genetic association loci. Clin Transl Gastroenterol.

[33] Singh T, Levine AP, Smith PJ, Smith AM, Segal AW, Barrett JC (2015). Characterization of expression quantitative trait loci in the human colon. Inflamm Bowel Dis.

[34] Kabakchiev B, Silverberg MS (2013). Expression quantitative trait loci analysis identifies associations between genotype and gene expression in human intestine. Gastroenterology.

[35] Wakil SM, Monies DM, Abouelhoda M, Al-Tassan N, Al-Dusery H, Naim EA (2015). Association of a mutation in LACC1 with a monogenic form of systemic juvenile idiopathic arthritis. Arthritis Rheumatol..

[36] Assadi G, Saleh R, Hadizadeh F, Vesterlund L, Bonfiglio F, Halfvarson J (2016). LACC1 polymorphisms in inflammatory bowel disease and juvenile idiopathic arthritis. Genes Immun.

[37] Kim D, Pertea G, Trapnell C, Pimentel H, Kelley R, Salzberg SL (2013). TopHat2: accurate alignment of transcriptomes in the presence of insertions, deletions and gene fusions. Genome Biol.

[38] Li H, Handsaker B, Wysoker A, Fennell T, Ruan J, Homer N, et al. 1000 Genome Project Data Processing Subgroup. The sequence alignment/map format and SAMtools. Bioinformatics 2009;25:2078–2079. 10.1093/bioinformatics/btp352 PMC2723002 19505943

[39] Anders S, Pyl PT, Huber W (2015). HTSeq—a Python framework to work with high-throughput sequencing data. Bioinformatics.

[40] Robinson MD, McCarthy DJ, Smyth GK (2010). edgeR: a Bioconductor package for differential expression analysis of digital gene expression data. Bioinformatics.

[41] Leek J, Johnson WE, Jaffe A, Parker H, Storey JD (2012). The SVA package for removing batch effects and other unwanted variation in high-throughput experiments. Bioinformatics.

[42] Mecham BH, Nelson PS, Storey JD (2010). Supervised normalization of microarrays. Bioinformatics.

[43] Young MD, Wakefield MJ, Smyth GK, Oshlack A (2010). Gene ontology analysis for RNA-seq: accounting for selection bias. Genome Biol.

[44] Preininger M, Arafat D, Kim J, Nath AP, Idaghdour Y, Brigham KL (2013). Blood-informative transcripts define nine common axes of peripheral blood gene expression. PLoS Genet.

[45] Li S, Rouphael N, Duraisingham S, Romero-Steiner S, Presnell S, Davis C (2014). Molecular signatures of antibody responses derived from a systems biological study of 5 human vaccines. Nat Immunol.

[46] JMP® Genomics, Version 8.0. SAS Institute Inc., Cary, NC, 1989–2015.

[47] Purcell S, Neale B, Todd-Brown K, Thomas L, Ferreira MAR, Bender D (2007). PLINK: a tool set for whole-genome association and population-based linkage analyses. Am J Hum Genet.

[48] Delaneau O, Coulonges C, Zagury JF (2008). Shape-IT: new rapid and accurate algorithm for haplotype inference. BMC Bioinformatics.

[49] Howie BN, Donnelly P, Marchini J (2009). A flexible and accurate genotype imputation method for the next generation of genome-wide association studies. PLoS Genet.

[50] Zhou X, Stephens M (2012). Genome-wide efficient mixed-model analysis for association studies. Nat Genet.

[51] Westra HJ, Peters MJ, Esko T, Yaghootkar H, Schurmann C, Kettunen J (2014). Systematic identification of trans eQTLs as putative drivers of known disease associations. Nat Genet.

[52] Favé MJ, Lamaze FC, Soave D, Hodgkinson A, Gauvin H, Bruat V (2018). Gene-by-environment interactions in urban populations modulate risk phenotypes. Nat Commun.

[53] Du N, Jiang K, Sawle AD, Frank MB, Wallace CA, Zhang A (2015). Dynamic tracking of functional gene modules in treated juvenile idiopathic arthritis. Genome Med..

[54] Hinze CH, Fall N, Thornton S, Mo JQ, Aronow BJ, Layh-Schmitt G (2010). Immature cell populations and an erythropoiesis gene-expression signature in systemic juvenile idiopathic arthritis: implications for pathogenesis. Arthritis Res Ther..

[55] Hu Z, Jiang K, Frank MB, Chen Y, Jarvis JN (2018). Modeling transcriptional rewiring in neutrophils through the course of treated juvenile idiopathic arthritis. Sci Rep.

[56] Giambartolomei C, Vukcevic D, Schadt EE, Franke L, Hingorani AD, Wallace C (2014). Bayesian test for colocalisation between pairs of genetic association studies using summary statistics. PLoS Genet.

[57] Pruim RJ, Welch RP, Sanna S, Teslovich TM, Chines PS, Gliedt TP (2010). LocusZoom: regional visualization of genome-wide association scan results. Bioinformatics.

[58] Liu JZ, van Sommeren S, Huang H, Ng SC, Alberts R, Takahashi A (2015). Association analyses identify 38 susceptibility loci for inflammatory bowel disease and highlight shared genetic risk across populations. Nat Genet.

[59] Okada Y, Wu D, Trynka G, Raj T, Terao C, Ikari K (2014). Genetics of rheumatoid arthritis contributes to biology and drug discovery. Nature.

[60] Marigorta UM, Denson LA, Hyams JS, Mondal K, Prince J, Walters TD (2017). Transcriptional risk scores link GWAS to eQTLs and predict complications in Crohn’s disease. Nat Genet.

[61] Chaussabel D, Quinn C, Shen J, Patel P, Glaser C, Baldwin N (2008). A modular analysis framework for blood genomics studies: application to systemic lupus erythematosus. Immunity.

[62] Gibson G, Powell JE, Marigorta UM (2015). Expression quantitative trait locus analysis for translational medicine. Genome Med..

[63] Ellinghaus D, Jostins L, Spain SL, Cortes A, Bethune J, Han B (2016). Analysis of five chronic inflammatory diseases identifies 27 new associations and highlights disease-specific patterns at shared loci. Nat Genet.

[64] Fairfax BP, Makino S, Radhakrishnan J, Plant K, Leslie S, Dilthey A (2012). Genetics of gene expression in primary immune cells identifies cell type-specific master regulators and roles of HLA alleles. Nat Genet.

[65] Peters JE, Lyons PA, Lee JC, Richard AC, Fortune MD, Newcombe PJ (2016). Insight into genotype-phenotype associations through eQTL mapping in multiple cell types in health and immune-mediated disease. PLoS Genet.

[66] Hemani G, Shakhbazov K, Westra HJ, Esko T, Henders AK, McRae AF (2012). Detection and replication of epistasis influencing transcription in humans. Nature.

[67] Mäki-Tanila A, Hill WG (2014). Influence of gene interaction on complex trait variation with multilocus models. Genetics.

[68] Newman AM, Liu CL, Green MR, Gentles AJ, Feng W, Xu Y (2015). Robust enumeration of cell subsets from tissue expression profiles. Nat Methods.

[69] Huang H, Fang M, Jostins L, Umićević Mirkov M, Boucher G (2017). Fine-mapping inflammatory bowel disease loci to single-variant resolution. Nature.

[70] Chun S, Casparino A, Patsopoulos NA, Croteau-Chonka DC, Raby BA, De Jager PL (2017). Limited statistical evidence for shared genetic effects of eQTLs and autoimmune-disease-associated loci in three major immune-cell types. Nat Genet.

[71] Tabassum R, Sivadas A, Agrawal V, Tian H, Arafat D, Gibson G (2015). Omic personality: implications of stable transcript and methylation profiles for personalized medicine. Genome Med..

[72] Carr EJ, Dooley J, Garcia-Perez JE, Lagou V, Lee JC, Wouters C (2016). The cellular composition of the human immune system is shaped by age and cohabitation. Nat Immunol.

[73] Lin YT, Wang CT, Gershwin ME, Chiang BL (2011). The pathogenesis of oligoarticular/polyarticular vs systemic juvenile idiopathic arthritis. Autoimmun Rev.

[74] McGonagle D, Aziz A, Dickie LJ, McDermott MF (2009). An integrated classification of pediatric inflammatory diseases, based on the concepts of autoinflammation and the immunological disease continuum. Pediatr Res.

[75] Jiang K, Wong L, Sawle AD, Frank MB, Chen Y, Wallace CA (2016). Whole blood expression profiling from the TREAT trial: insights for the pathogenesis of polyarticular juvenile idiopathic arthritis. Arthritis Res Ther..

[76] Idaghdour Y, Storey JD, Jadallah SJ, Gibson G (2008). A genome-wide gene expression signature of environmental geography in leukocytes of Moroccan Amazighs. PLoS Genet.

[77] Banchereau R, Hong S, Cantarel B, Baldwin N, Baisch J, Edens M (2016). Personalized immunomonitoring uncovers molecular networks that stratify lupus patients. Cell.

