# Supplementary material for: Disease-specific regulation of gene expression in a comparative analysis of juvenile idiopathic arthritis and inflammatory bowel disease
Source: Genome Med. 2018 Jun 27;10:48. doi: 10.1186/s13073-018-0558-x (PMC6020373; doi:10.1186/s13073-018-0558-x)

Figure S1. Effects of medication and sample time on gene expression. Changes in gene expression following adjustment for treatment with DMARDs, biologics, and steroids, as well as time from diagnosis to sample collection. From left to right in pairs, (a) depicts the principal component of unadjusted expression of Axis genes versus adjusted expression. (b) depicts the principal component of time to collection adjusted expression by days to sample collection.

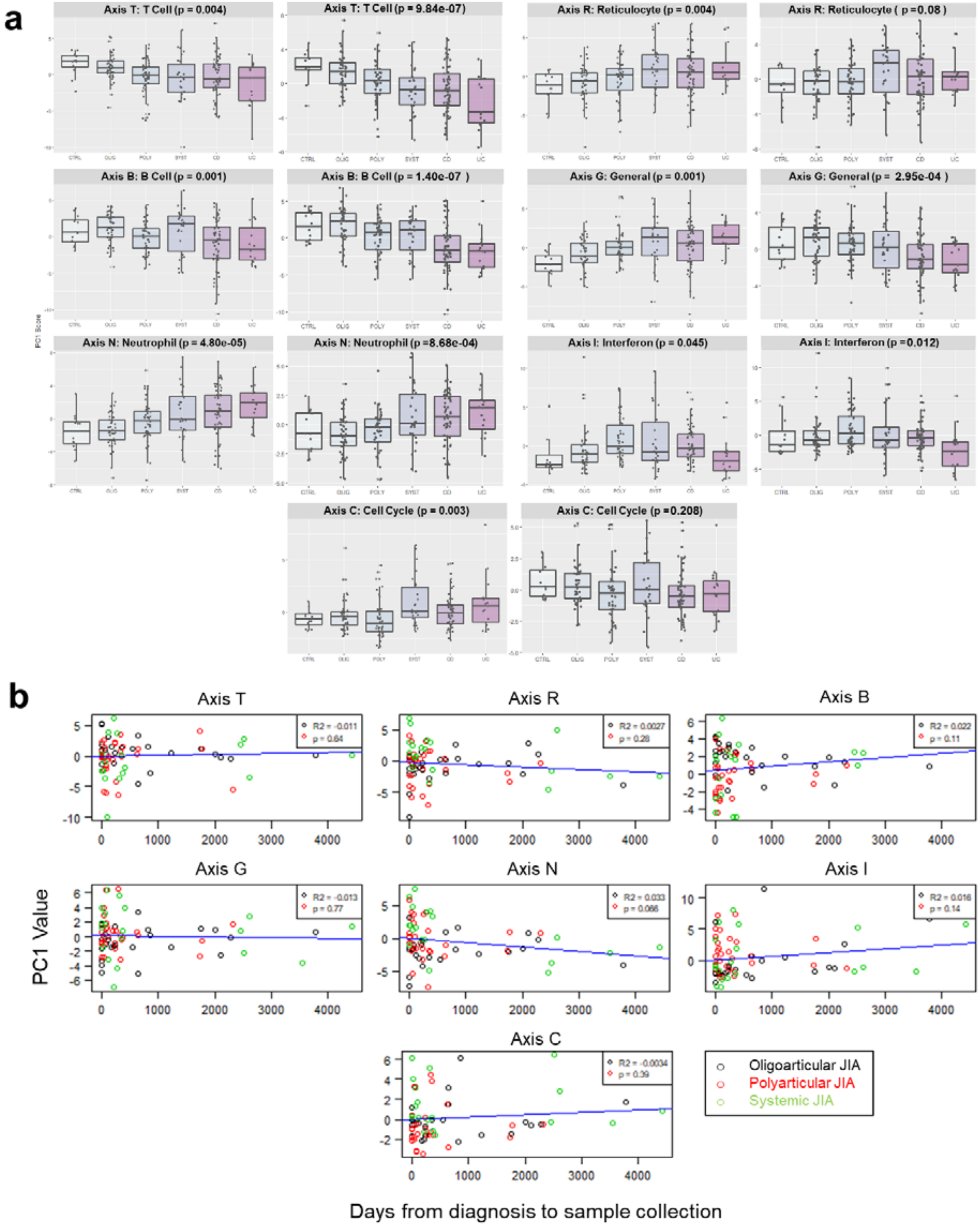

Figure S2. Correlation of betas in non-adjusted and medication-adjusted SNPs. Effect sizes in DMARD, biologic, and steroid treatment-adjusted SNPs versus non-adjusted SNPs. SNPs highlighted in red are significant at $p < 0.0001$, with a correlation = 0.99. All SNPs have a correlation of 0.82.

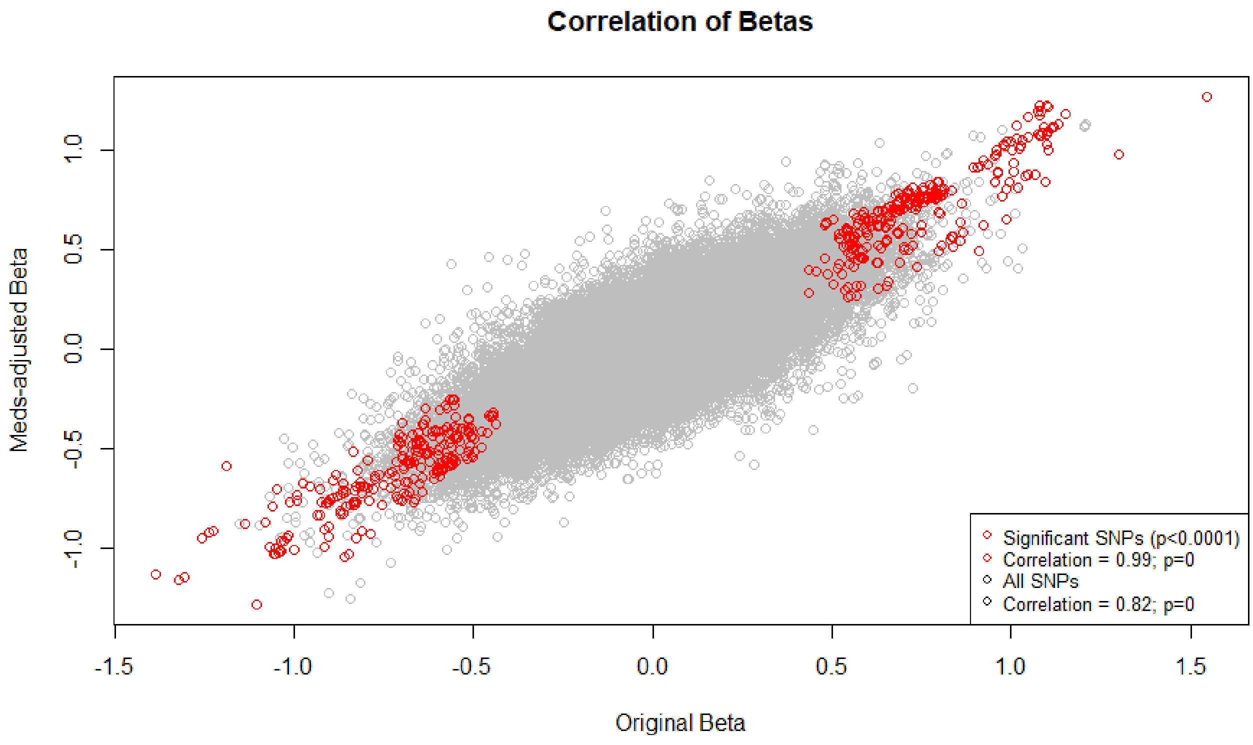

Figure S3. Replication of gene expression trends in the Hinze et al. dataset. The principal component of Axis gene expression in the dataset presented in this analysis, versus the principal component derived from the Hinze et al. dataset.

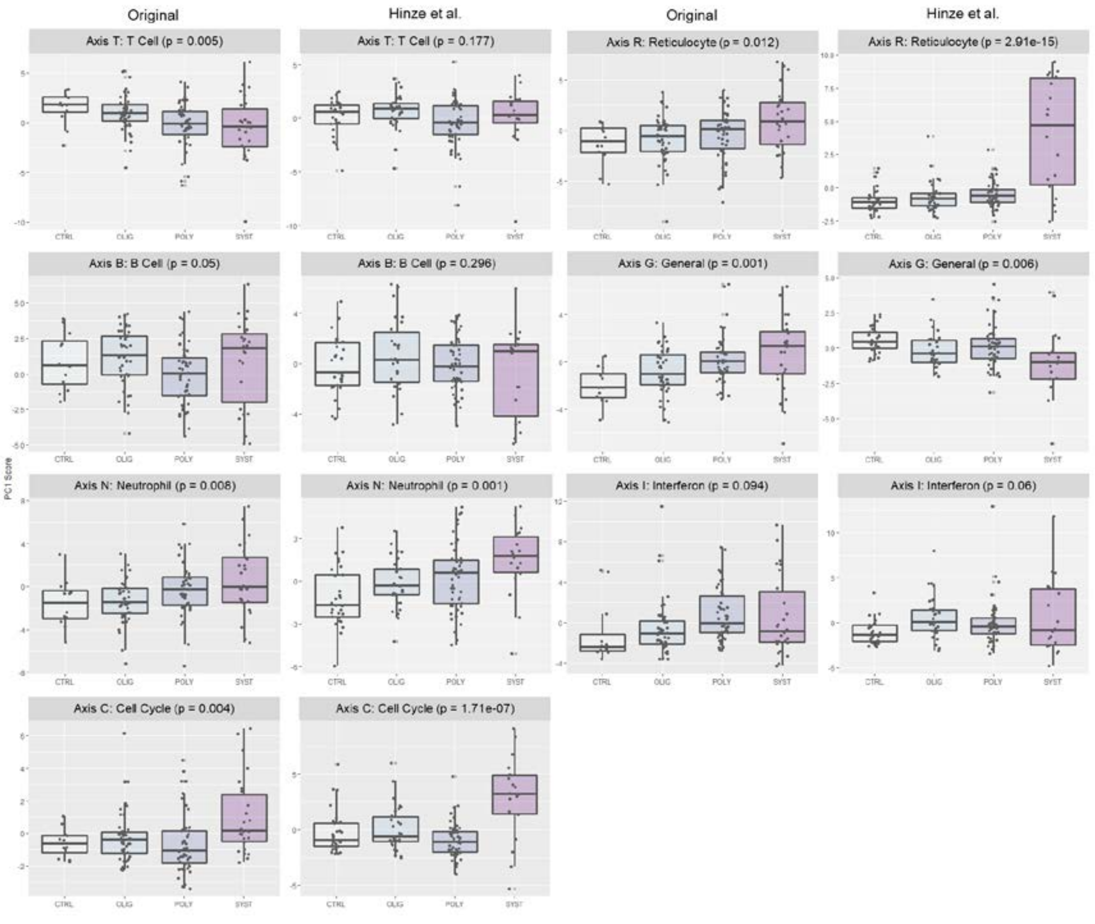

Figure S4. Examples of disease-specific eQTL. Selection of eQTL with JIA- or IBD-biased effects on transcript abundance. The p-values for presence of an interaction effect between genotype and disease are noted in headers.

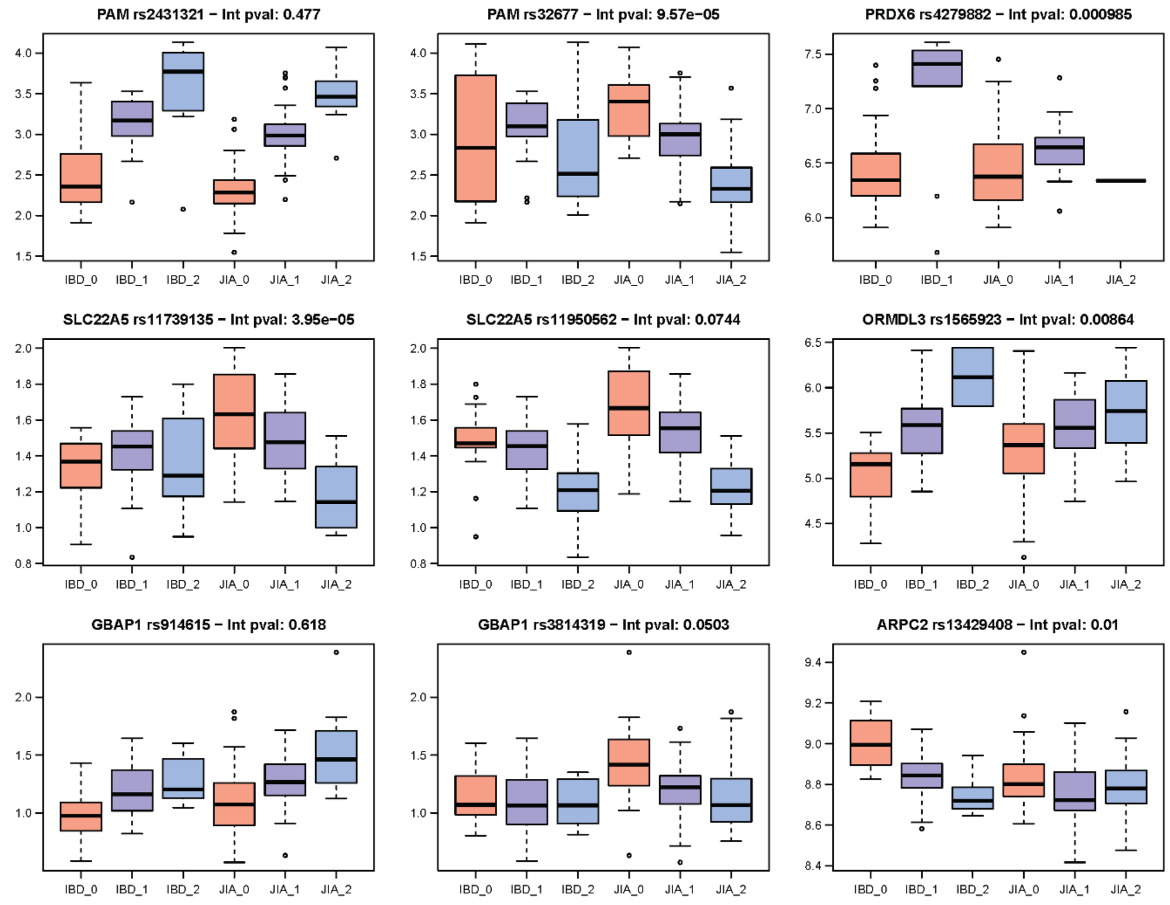

Figure S5. Interaction effects with addition of ethnicity. The p-value for presence of interaction effects in a model including ancestry, versus p-value in a model without. The genotype-disease interaction effects remain significant even after addition of the ethnicity term.

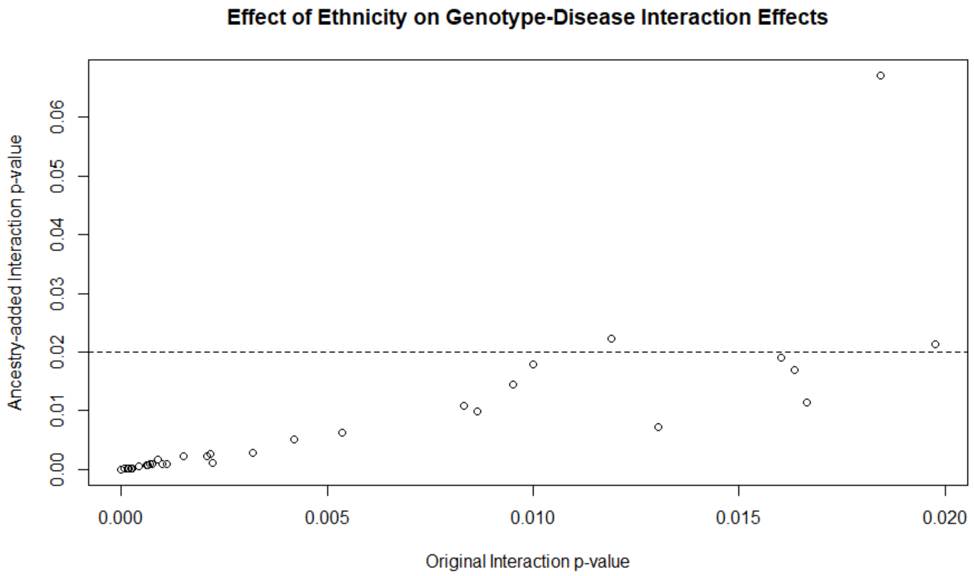

Supplement: Supplementary file 1 — Figure S1. Effects of medication and sample time on gene expression. Figure S2. Correlation of betas in non-adjusted and medication-adjusted SNPs. Figure S3. Replication of gene expression trends in the Hinze et al. dataset. Figure S4. Examples of disease-specific eQTL. Figure S5. Interaction effects with addition of ethnicity. (PDF 630 kb) [file 13073_2018_558_MOESM1_ESM.pdf]
